# Myocardial Infarction with Nonobstructive Coronary Arteries (MINOCA): Current Insights into Pathophysiology, Diagnosis, and Management

**DOI:** 10.3390/diagnostics15070942

**Published:** 2025-04-07

**Authors:** Chiara Tognola, Alessandro Maloberti, Marisa Varrenti, Patrizio Mazzone, Cristina Giannattasio, Fabrizio Guarracini

**Affiliations:** 1 Clinical Cardiology Unit, De Gasperis Cardio Center, Niguarda Hospital, 20162 Milan, Italy; alessandro.maloberti@ospedaleniguarda.it (A.M.); cristina.giannattasio@ospedaleniguarda.it (C.G.); 2 School of Medicine and Surgery, University of Milano-Bicocca, 20126 Milan, Italy; 3 Electrophysiology Unit, De Gasperis Cardio Center, Niguarda Hospital, 20162 Milan, Italy; marisa.varrenti@ospedaleniguarda.it (M.V.); patrizio.mazzone@ospedaleniguarda.it (P.M.); fabrizio.guarracini@ospedaleniguarda.it (F.G.)

**Keywords:** MINOCA, myocardial infarction, nonobstructive coronary arteries, pathophysiology, diagnosis, cardiac magnetic resonance, coronary microvascular dysfunction, coronary vasospasm, sex differences, management

## Abstract

Myocardial infarction with nonobstructive coronary arteries (MINOCA) is an increasingly recognized clinical entity characterized by myocardial injury in the absence of a significant coronary artery obstruction. MINOCA encompasses a diverse range of pathophysiological mechanisms, including coronary plaque disruption, coronary vasospasm, coronary microvascular dysfunction, thromboembolism, and spontaneous coronary artery dissection. A systematic diagnostic approach is essential to identify the underlying etiology and guide appropriate management strategies. Advanced imaging techniques, particularly cardiac magnetic resonance, play a pivotal role in distinguishing ischemic from non-ischemic myocardial injury and refining prognosis. Despite growing awareness, standardized treatment protocols remain limited, with current management largely extrapolated from strategies used in obstructive coronary artery disease. Notably, MINOCA is significantly more prevalent in women, emphasizing the need to understand sex-related differences in its pathophysiology, presentation, and clinical outcomes. This narrative review offers a comprehensive and up-to-date overview of MINOCA, including a dedicated chapter on sex-related considerations. It integrates recent advancements and highlights the importance of personalized management strategies.

## 1. Introduction

Myocardial infarction with nonobstructive coronary arteries (MINOCA) is a clinical syndrome in which patients present with myocardial injury despite the absence of hemodynamically significant coronary artery stenosis (stenosis < 50%) on angiography [1]. This heterogeneous condition includes diverse pathophysiological mechanisms leading to myocardial ischemia and necrosis without a significant coronary obstruction. MINOCA can be categorized into cases with no or minimal atherosclerosis (0–30% stenosis) and those with mild atherosclerosis (30–49% stenosis) [2]. Due to its diverse etiologies, MINOCA should be considered with an operational diagnosis requiring a systematic approach to determine the underlying etiology. The diagnosis of MINOCA is established when patients meet the criteria for acute myocardial infarction (MI), as defined by the Fourth Universal Definition of Myocardial Infarction, while ruling out obstructive coronary artery disease [3]. Despite its increasing recognition, MINOCA remains underdiagnosed and frequently misclassified due to the complexities involved in identifying its underlying causes. Epidemiological data suggest that MINOCA disproportionately affects postmenopausal women [4]. Although it can result from coronary plaque disruption, coronary spasm, coronary microvascular dysfunction (CMD), thromboembolism, and spontaneous coronary artery dissection (SCAD), emerging evidence suggests that women are more frequently affected by CMD and SCAD, whereas men are more likely to have thromboembolic or atherosclerotic causes [5]. Given the increasing recognition of MINOCA, a deeper understanding of its pathophysiological mechanisms is crucial for improving diagnostic accuracy and clinical management. This narrative review provides a comprehensive examination of the epidemiology, pathophysiology, clinical presentation, diagnostic modalities, and management strategies for MINOCA.

## 2. Epidemiology and Prognosis

MINOCA accounts for approximately 5% to 10% of all acute myocardial infarction cases. Meta-analyses estimate that it constitutes around 6% of all myocardial infarctions across various populations [6]. Demographically, MINOCA patients tend to be younger than those with myocardial infarction due to obstructive coronary artery disease (MI-CAD). Women comprise more than half of MINOCA cases, in contrast to MI-CAD, where men predominate [7]. Ethnic variations have also been observed, with higher prevalence rates reported among Black, Māori, Pacific Islander, and Hispanic individuals. Once considered to be a benign condition, MINOCA is now recognized as carrying a significant long-term cardiovascular risk. Studies report an annual mortality rate of approximately 2%, with an increased risk of recurrent myocardial infarction, heart failure, and stroke [8,9]. Elevated peak cardiac troponin levels correlate with long-term adverse outcomes [10]. A systematic review found that the 12-month all-cause mortality for suspected MINOCA patients was 3.4%, with a major adverse cardiovascular event (MACE) rate of 9.6% [11]. Furthermore, prognosis varies by sex and age, with older individuals and women facing a higher risk of heart failure and long-term cardiovascular events, including mortality and hospital readmission [12].

## 3. Pathophysiology

The mechanisms underlying MINOCA are diverse and include both atherosclerotic and non-atherosclerotic causes. Understanding these mechanisms is essential for accurate diagnosis and effective treatment.

### 3.1. Atherosclerotic Causes

- Coronary plaque disruption

Plaque rupture, plaque erosion, or calcified nodules may lead to luminal thrombus formation without significant coronary stenosis.

Plaque rupture, the most common cause of coronary thrombosis in men, occurs when a defect in the fibrous cap exposes the lipid-rich core of the atherosclerotic plaque to the bloodstream, triggering thrombus formation. This process is associated with fibrous cap thinning due to (i) the depletion of vascular smooth muscle cells (VSMCs) within the fibrous cap and (ii) macrophage infiltration, leading to degradation of the collagen-rich cap matrix [13]. Plaque erosion, the second leading cause of coronary thrombosis, involves endothelial cell loss or dysfunction without a visible structural defect. Endothelial cell apoptosis and detachment from the extracellular matrix are believed to contribute to this process [14]. Vasospasm has also been suggested to be a contributor to endothelial damage and subsequent thrombosis [15]. Calcified nodules, a less common cause of coronary thrombosis, occur when dysfunctional or sparse endothelial cells overlying these nodules increase thrombosis risk. Intracoronary imaging, such as optical coherence tomography (OCT) or high-definition intravascular ultrasound (IVUS), is required for a definitive diagnosis. An OCT-based study by Reynolds et al. found culprit lesions in 46.2% of women with MINOCA, with plaque erosion being the most common finding [16]. Smaller studies have also demonstrated the utility of OCT in detecting etiologic plaques in suspected MINOCA cases [17,18].

### 3.2. Non-Atherosclerotic Causes

- Coronary Artery Spasm

Coronary artery spasm can occur in both epicardial arteries or the coronary microvasculature. Epicardial coronary spasm is a transient near-total or complete occlusion (≥90% constriction) of a large coronary artery, causing angina and ischemic electrocardiographic changes, either occurring spontaneously or in response to a provocative stimulus. Vasospastic angina (VSA), also known as Prinzmetal angina, is a clinical syndrome defined by (i) typical symptoms of vasospasm, (ii) documented myocardial ischemia during spontaneous episodes, and (iii) confirmed coronary artery spasm. Provocative testing is often necessary to confirm heightened vasoreactivity and is recommended for patients with suspected VSA (class I) [19]. Identifying vasospasm is crucial as it necessitates specific therapeutic strategies, including the use of calcium channel blockers and nitrates to prevent recurrent episodes [20]. Epicardial spasm is associated with endothelial dysfunction and heightened VSMC reactivity [21]. A meta-analysis encompassing 25 studies with 14,554 patients found a higher prevalence in men (61%).

- Coronary Microvascular Dysfunction

CMD is diagnosed based on the following four criteria: (i) myocardial-ischemia-related symptoms, (ii) an absence of obstructive CAD, (iii) objective evidence of myocardial ischemia, and (iv) impaired coronary microvascular function. CMD can be identified through (i) a reduced coronary flow reserve (CFR) of less than 2.0 in response to a vasodilator, assessed via Doppler or thermodilution techniques; (ii) increased microvascular resistance, indicated by an index of microvascular resistance (IMR) exceeding 25; (iii) the coronary slow-flow phenomenon, defined as a thrombolysis in myocardial infarction (TIMI) frame count greater than 25; or (iv) microvascular spasm confirmed by provocative testing [22]. The gold standard for diagnosing CMD is invasive coronary function testing, which is advised for patients experiencing persistent angina despite nonobstructive coronary findings (class 2a recommendation) [23]. Non-invasive imaging alternatives include positron emission tomography (PET), stress cardiac magnetic resonance imaging, and stress echocardiography with coronary flow velocity reserve measurement [23,24]. CMD can result from either functional, such as impaired vasodilation, or excessive vasoconstriction, or a combination of both [24,25,26,27,28].

- Coronary embolism and thrombosis

Coronary thromboembolism can be classified into the following three types: direct, iatrogenic, and paradoxical [29]. Direct embolism originates from embolic material dislodged from left-sided cardiac structures, including mural thrombi, infectious endocarditis, or intracardiac tumors such as myxomas [30]. Individuals with underlying cardiac conditions, such as cardiomyopathies, atrial fibrillation, valvular disease, or coronary artery ectasia, have a heightened risk of direct embolism due to blood stagnation and reduced flow [29]. Although rare, infective endocarditis can also lead to coronary embolism, particularly in cases involving mitral valve infection, *Staphylococcus aureus* or fungal pathogens, and vegetations exceeding 10 mm in size [31]. Additionally, nonbacterial thrombotic endocarditis, or marantic endocarditis, has been reported to be an infrequent cause of embolism [32]. Among cardiac tumors, myxomas with a villiform texture are more prone to embolization than those with smooth surfaces [30]. Iatrogenic coronary embolism typically occurs during coronary interventions but can also arise in the context of cardiac surgery or valvular procedures [29]. In contrast, paradoxical embolism occurs when embolic material from the systemic venous circulation bypasses pulmonary filtration due to a right-to-left shunt, such as a patent foramen ovale or atrial septal defect [33]. In situ coronary thrombosis may also be associated with inherited or acquired hypercoagulable disorders, the most frequent etiology among female populations. A genetic predisposition to thrombosis was identified in 14% of MINOCA patients undergoing thrombophilia screening post-infarction, with abnormalities including factor V Leiden mutations, deficiencies in proteins C and S, and prothrombin gene mutations [6,34]. Acquired prothrombotic states that increase the risk of coronary thrombosis include conditions such as thrombotic thrombocytopenic purpura, antiphospholipid syndrome, heparin-induced thrombocytopenia, and myeloproliferative neoplasms [35]. In a prospective study of 84 MINOCA patients, 15.5% were diagnosed with antiphospholipid syndrome [36].

- Spontaneous Coronary Artery Dissection

SCAD is the separation of the coronary artery wall layers, leading to intramural hemorrhage without atherosclerosis or external injury [2]. It is increasingly recognized as a cause of acute myocardial infarction, necessitating a high level of suspicion, particularly in young women [37,38]. SCAD-related obstruction arises from the separation of the media and adventitia, leading to intramural hematoma formation. The “outside-in” hypothesis posits that the primary event is a vasa vasorum rupture, whereas the “inside-out” hypothesis suggests an intimal tear as the initial trigger [39,40]. SCAD may arise due to an underlying vasculopathy triggered by various stressors, such as catecholamine surges, emotional stress, intense physical exertion, sympathomimetic drug use, or hormonal changes [37,40]. Fibromuscular dysplasia was identified in 72% of patients from a cohort of 168 individuals with SCAD, with approximately half reporting a physical or emotional trigger [37]. The presence of coronary artery tortuosity (e.g., corkscrew appearance or multivessel symmetrical tortuosity) should raise suspicion for SCAD. To confirm the diagnosis, intravascular imaging techniques such as OCT and IVUS may be required to reveal the absence of significant atherosclerotic plaque and the presence of a dissection or intramural hematoma. OCT, with its higher spatial resolution, is often the preferred imaging method in suspected SCAD cases, though it should be used cautiously to avoid exacerbating the dissection by high-pressure contrast injection [41].

- Myocardial bridging

Myocardial bridging, recently identified as a non-atherosclerotic cause of MINOCA, occurs when a segment of a coronary artery is compressed during systole. This can cause transient ischemic episodes, particularly under conditions of increased myocardial demand [42]. Functional assessments using IVUS and FFR help to confirm the diagnosis.

### 3.3. Supply–Demand Mismatch

Supply–demand mismatch, known as type II MI, may lead to MINOCA. Approximately 50% of patients with type II MI do not have significant CAD, and they can be classified as MINOCA [4]. Stressors that reduce oxygen supply, like severe bradyarrhythmia, respiratory failure with profound hypoxemia, or significant anemia, can trigger MINOCA. Conversely, stressors that increase oxygen demand, such as sustained tachyarrhythmia, extreme hypertension, or thyrotoxicosis, can also contribute [2]. In patients with stable CAD, an acute stress event causing ischemic symptoms may lead to MINOCA due to the inability of the heart to meet increased oxygen demands or further compromise the supply caused by the stressor.

### 3.4. MINOCA Mimics

Several non-ischemic conditions can mimic MINOCA. A study by Reynolds et al. found that 20.7% of suspected MINOCA cases had non-ischemic patterns on CMR imaging, including myocarditis, takotsubo syndrome (TTS), or non-ischemic cardiomyopathy [16].

**Figure 1:**
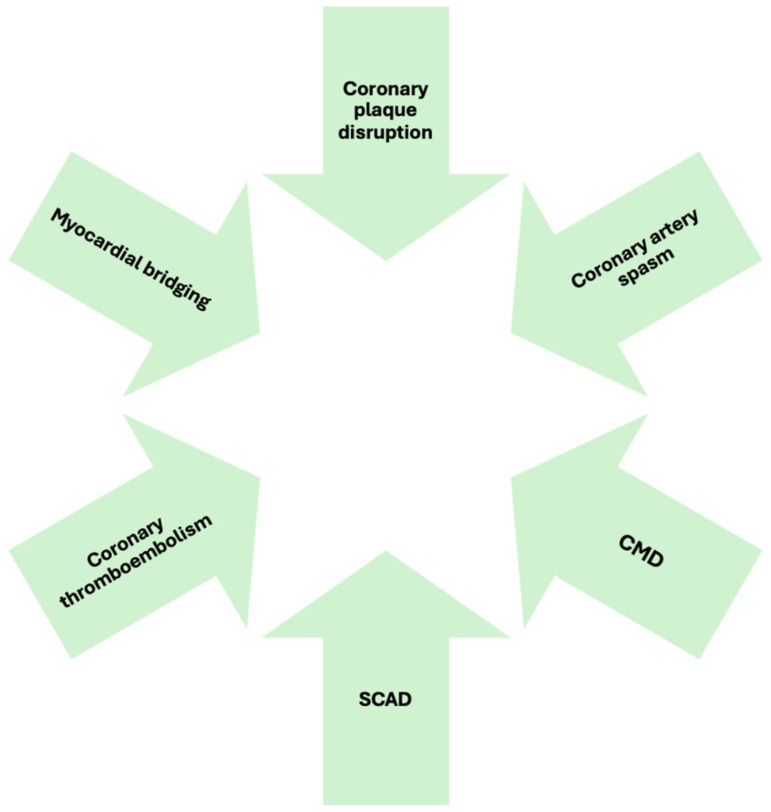
MINOCA pathophysiological mechanisms.

Figure 1 illustrates pathophysiological mechanism of MINOCA.

## 4. Clinical Presentation

Patients with MINOCA commonly report chest pain that mimics classic angina, often described as a pressure-like or constrictive discomfort in the chest, radiating to the left arm, jaw, or back. This pain may be triggered by exertion or emotional stress and relieved by rest or nitroglycerin [43]. However, many patients present with atypical symptoms, including nausea, diaphoresis, epigastric discomfort, vomiting, dizziness, dyspnea, or profound fatigue. These non-specific symptoms frequently lead to misdiagnosis or a delay in seeking medical attention as they are often attributed to non-cardiac conditions such as anxiety, musculoskeletal disorders, or gastrointestinal conditions. Dyspnea is a common complaint, especially in microvascular dysfunction. Some patients experience palpitations, which may be indicative of concurrent arrhythmic activity, particularly in cases of coronary spasm. Electrocardiography (ECG) findings vary depending on the underlying pathophysiology. Most patients present with non-ST-elevation myocardial infarction (NSTEMI). A recent study of over 3000 MINOCA patients found that 83.9% presented with NSTEMI, while 16.1% presented with STEMI [44]. ECG may also reveal non-specific T-wave abnormalities. ST-segment elevation is more commonly associated with coronary spasm, whereas patients with microvascular dysfunction often exhibit T-wave inversions or subtle ischemic changes. In some cases, the ECG findings are normal at presentation. The presence of transient ECG abnormalities, such as dynamic ST-segment changes, suggests an underlying vasospastic component [16]. The degree of troponin elevation varies, with lower peak levels often observed in cases driven by microvascular dysfunction rather than epicardial coronary occlusion. The inconsistency between symptom severity, ECG findings, and troponin levels often raises diagnostic uncertainty, necessitating further imaging and functional testing to establish the underlying etiology [6]. Beyond confirming myocardial injury, recent evidence suggests that elevated troponin T (TnT) levels provide prognostic insights in MINOCA. Studies have shown that persistently high TnT values are associated with an increased risk of adverse cardiovascular events, including heart failure and mortality [45].

## 5. Diagnosis

The European Society of Cardiology (ESC) was the first international organization to establish formal diagnostic criteria for MINOCA [1]. Their proposed definition consists of the following three key components: (i) fulfillment of the criteria for acute myocardial infarction as outlined in the Third Universal Definition of Myocardial Infarction [1]; (ii) confirmation of nonobstructive coronary arteries based on an angiographic assessment, ensuring that no major epicardial coronary vessel has stenosis exceeding 50%; and (iii) exclusion of other overt clinical conditions that could account for the acute presentation. A fundamental aspect of this definition is the presence of myocardial injury, as evidenced by elevated cardiac troponin levels above the 99th percentile upper reference limit, with a characteristic rise and fall on serial testing. However, although troponin elevation indicates myocardial injury, it is not specific to ischemia, as non-ischemic conditions also lead to similar biomarker elevations. Recognizing this limitation, the Fourth Universal Definition of Myocardial Infarction, developed by a joint task force of the ESC, American College of Cardiology (ACC), American Heart Association (AHA), and World Heart Federation (WHF), introduced a revised framework to distinguish myocardial injury from myocardial infarction [3]. Accordingly, the term MINOCA should be exclusively reserved for cases where an ischemic origin is identified as the cause of the clinical presentation. This necessitates a meticulous diagnostic approach when evaluating patients with suspected MI who lack significant coronary artery stenosis. To establish a diagnosis of MINOCA, clinicians must systematically rule out extracardiac causes of myocardial injury such as sepsis, pulmonary embolism, or aortic dissection through an evaluation of the clinical context, laboratory tests, and echocardiography. Subsequently, CMR can help to rule out cardiac causes of myocardial injury, such as myocarditis, TTS, and cardiomyopathies, and confirm myocardial infarction. Once these possibilities have been systematically excluded, a definitive diagnosis of MINOCA can be established. To further define the underlying etiology, intravascular imaging (OCT and IVUS), intracoronary functional tests (Ach/ergonovine), and coronary physiology assessments (FFR, CMR, and IMR) are necessary.

CMR is highly recommended as a critical tool in diagnosing MINOCA as it can rule out conditions like myocarditis, TTS, and cardiomyopathies, while confirming MI. Recent evidence highlights the importance of distinguishing MINOCA mimics from true MINOCA as they have differing prognoses. A recent meta-analysis demonstrated that conditions such as myocarditis and takotsubo syndrome, often mistaken for MINOCA, have distinct long-term outcomes. Although true MINOCA is associated with an increased risk of recurrent cardiovascular events, MINOCA mimics may present with a lower incidence of MACE but still require careful risk stratification [46]. The reported prevalence of TTS and myocarditis in patients diagnosed with MINOCA via CMR varies significantly in the literature, ranging from 2% to 27% for TTS and 7% to 63% for myocarditis [6,18,47,48]. These differences can be attributed to factors such as the selection criteria for study participants, the timing of the CMR assessment, and the specific CMR techniques and diagnostic criteria employed. CMR with LGE enables the evaluation of the infarct location and size. However, in a significant proportion of cases (ranging from 8% to 73%) where myocarditis or TTS is excluded by CMR, a localized infarct is not detected by LGE [6,18,47,48,49]. Contemporary LGE imaging, using sequences with an average voxel size of 1.4 × 1.8 × 6 (−8) mm, requires a minimum of 0.2 g of infarcted myocardial tissue to be visible [50]. This threshold is higher than the amount of damaged myocardium needed to cause small but notable increases in cardiac troponin levels, which may account for the lack of visible infarction on CMR in many MINOCA patients. Nonetheless, it remains possible that the elevated troponin levels in these cases could be due to factors other than ischemic necrosis. Standard CMR protocols provide a comprehensive evaluation of both cardiac anatomy and function, utilizing steady-state free precession (SSFP) MRI or cine imaging. They also assess tissue properties, with T2-weighted imaging sequences (T2w) and T2-weighted fat-suppressed sequences (T2wSTIR) being valuable for identifying localized edema. Additionally, T1-weighted imaging following LGE helps to detect areas of acute cellular membrane damage or chronic myocardial scarring. The distribution of LGE can differentiate between myocardial infarction, which typically shows subendocardial or transmural enhancement, and myocarditis, which often exhibits a subepicardial or mid-wall pattern. TTS typically presents with apical and mid-ventricular edema accompanied by transient dyskinesia in these regions, while the basal segments exhibit hyperkinesia and no LGE is observed. Advancements in MRI technology have enhanced diagnostic accuracy. Parametric T1-mapping (which calculates extracellular volume (ECV)), along with T2-mapping sequences, allows for a precise pixel-by-pixel analysis of myocardial tissue characteristics. This approach assesses both extracellular and intracellular features, such as water content, which can indicate inflammation or edema. Moreover, T1- and T2-mapping can detect diffuse changes, like generalized fibrosis, which are not visible with LGE [51]. The updated Lake Louise Criteria for CMR in myocardial inflammation incorporate mapping sequences, refining the imaging diagnosis by considering both T1 criteria (e.g., the presence of LGE or increased T1-mapping or ECV values) and T2 criteria (e.g., hyperintensity in T2wSTIR or elevated T2-mapping values) [52]. The introduction of free-breathing LGE techniques has notably improved the spatial resolution, making it five times more effective and allowing for the detection of smaller areas of necrosis. In a recent study, incorporating this enhanced sequence into standard imaging protocols enabled a definitive diagnosis in 48% of MINOCA patients who initially had normal scans [53]. The diagnostic accuracy of CMR is maximized when performed within 7 to 14 days of symptom onset. Delayed imaging can lead to the disappearance of certain pathological markers, such as myocardial edema in myocarditis. A study by Dastidar et al. compared retrospective scans of MINOCA patients and found that early CMR (within two weeks of admission) reduced inconclusive results from 43% to 16% [54]. This improvement was primarily due to the better detection of takotsubo syndrome and myocarditis. In patients with a MINOCA diagnosis, serial CMR scans revealed that myocardial edema and epicardial LGE patterns resolved in about one-quarter of cases within three weeks of admission.

Beyond its diagnostic utility, CMR has significant prognostic value in MINOCA. Recent findings suggest that different CMR-defined phenotypes of ischemic myocardial injury correlate with varying long-term outcomes [55]. The presence of LGE in a subendocardial or transmural distribution is associated with a higher risk of MACE, whereas patients with no LGE or with non-ischemic patterns tend to have a more favorable prognosis. These data underscore the importance of incorporating CMR in the risk stratification process for MINOCA patients.

Invasive testing includes intravascular imaging (OCT and IVUS), intracoronary functional testing (Ach/ergonovine), and coronary physiology assessments (FFR, CMR, and IMR).

- Intravascular imaging (OCT and IVUS)

OCT, a high-resolution imaging technique, characterizes coronary plaque components (fibrous cap, thrombus, and calcification) and vessel walls (intima, media, and adventitia). OCT offers a better resolution (15–20 µm) than angiography. Although its role in MINOCA remains underexplored, studies have shown its effectiveness in detecting plaque rupture and thrombi. Yamamoto et al. (2019) reported that OCT identified abnormal findings in 25% of MINOCA patients with ischemic symptoms but angiographically nonobstructive lesions, including thrombi and plaque rupture [56]. Taruya et al. (2016) demonstrated OCT’s prognostic value, finding hidden high-risk lesions in 51% of MINOCA patients. During follow-up, 10% of patients with these high-risk lesions developed ACS [57]. Similarly, a study by Mas-Lladò et al. (2020) revealed that OCT identified atherosclerotic plaques and spontaneous coronary dissection [58]. A larger study by Zeng et al. (2022) found that 33.7% of MINOCA cases had atherosclerotic causes, with worse clinical outcomes compared with non-atherosclerotic causes, such as spontaneous dissection or coronary spasm [59]. Patients with atherosclerotic MINOCA had more adverse events, including major cardiac events, revascularizations, and rehospitalizations. In the context of SCAD, OCT provides valuable information to assess the length of intramural hematomas, the extent of luminal compromise, and the thickness of a dissected tear. However, the risk of worsening the false lumen during contrast injection should be considered [60]. Limitations of OCT are related to the evaluation of the proximal part of the left main or right coronary artery or in the case of coronary ectasia. IVUS provides deeper tissue penetration (4–8 mm versus OCT’s 1–3 mm), making it effective for exploring outer plaque layers and assessing lipid content and vessel remodeling. IVUS is also better at visualizing calcified lesions and does not require contrast administration, making it the preferred option for patients with chronic kidney disease. IVUS is well-established for managing obstructive CAD during percutaneous revascularization but its role in MINOCA management remains underexplored.

- Intracoronary functional test (Ach/ergonovine)

Intracoronary acetylcholine testing is currently the preferred method to evaluate coronary epicardial and microvascular endothelium-dependent vasodilation and vasospasm. It involves intracoronary acetylcholine boluses (20–200 mcg) and subsequent assessments of the coronary response using invasive contrast angiography. The test is considered to be positive for macrovascular spasm if symptoms occur, accompanied by ischemic ECG changes and an angiographic reduction of ≥90% of the coronary lumen [61]. If the lumen reduction is <90%, a diagnosis of microvascular spasm is made. The vasospastic effect of Ach is rapidly transient and can, if needed, be reversed by the intracoronary administration of nitroglycerine, which also allows an assessment of endothelium-independent epicardial coronary vasodilation. Large registries have demonstrated that an intracoronary acetylcholine bolus up to a maximum of 200 mcg in stable patients has a sustainable safety profile [62,63]. It should be performed at least 24 h after a wash-out from CCB and nitrates. The use of intracoronary ergonovine boluses (20–60 mcg) is a possible alternative.

- Coronary physiology assessments (FFR, CMR, and IMR)

To assess flow-limiting obstructive coronary artery disease, FFR is used. FFR is the ratio of distal coronary pressure to aortic pressure at maximal hyperemia, with abnormal FFR values defined as ≤0.80 or a non-hyperemic pressure ratio of ≤0.89. These values must be interpreted within the context of the individual patient’s clinical situation. Data from stable patients suggest that up to a quarter of those with 30%–50% stenosis show functionally significant lesions when assessed using FFR. If FFR is utilized, it is recommended that only those with an FFR >0.80 be included as part of a MINOCA diagnosis [64]. Studies typically use a CFR cut-off of 2.0 for thermodilution or 2.5 for Doppler flow velocity as markers of prognostic significance [65,66]. Microvascular resistance is assessed by combining pressure and flow data. The index of microvascular resistance (IMR) is calculated as the product of distal coronary pressure during maximal hyperemia and the hyperemic mean transit time. An IMR ≥25 indicates microvascular dysfunction. Alternatively, the hyperemic myocardial velocity resistance (HMR) index is based on the Doppler flow, calculated by dividing intracoronary pressure by hyperemic flow velocity. Research has shown that an HMR >1.9 is a strong predictor of recurrent chest pain in patients with angina and nonobstructive coronary arteries. Together, CFR, IMR, and FFR provide comprehensive diagnostic information regarding endothelial-independent CMD, endothelium-dependent microvascular function, vasospastic responses, and low-grade stenoses. CMD is characterized by a decreased CFR and increased microvascular resistance (IMR and HMR).

**Figure 2:**
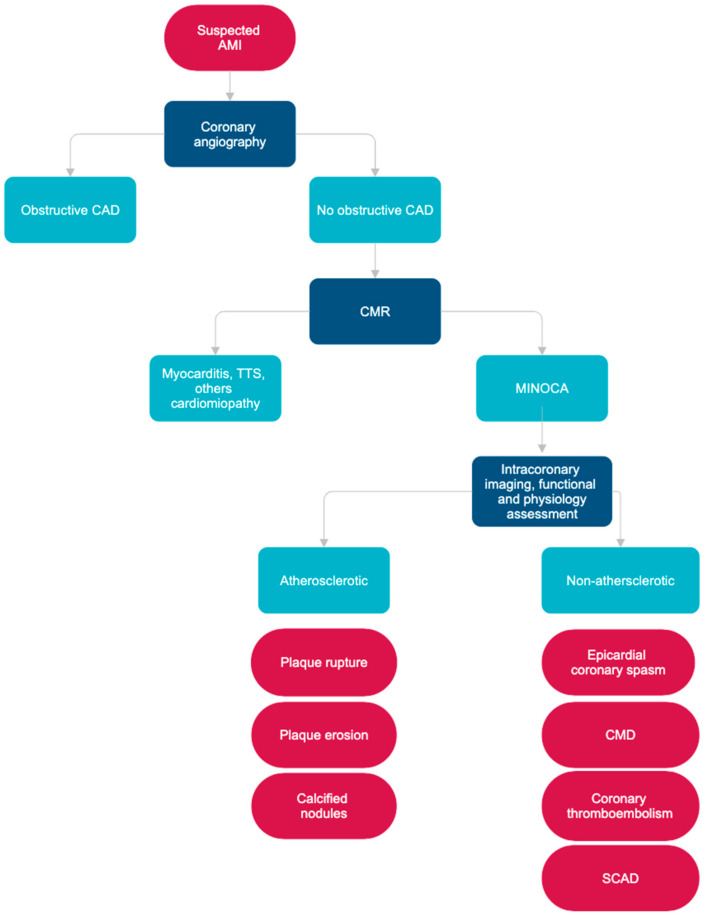
Clinical algorithm for the diagnosis of MINOCA.

Figure 2 presents a clinical algorithm to diagnose MINOCA.

## 6. Management

Currently, no randomized clinical trials have been published on the treatment of MINOCA. As a result, existing guidelines primarily rely on expert consensus rather than high-quality evidence. The complexity of MINOCA as a heterogeneous condition further complicates treatment decisions as the precise underlying cause is often undetermined in routine clinical practice. Acute-phase management primarily focuses on symptom relief as no intervention has been demonstrated to limit the infarct size in MINOCA. Long-term low-dose aspirin is generally recommended for secondary prevention [2]. However, the role of DAPT remains controversial, with no observational studies indicating a significant reduction in cardiovascular risk [67,68,69,70,71]. Conversely, statin therapy has been associated with lower mortality and reduced MACE, with effects comparable to those observed in randomized trials of secondary prevention in traditional myocardial infarction [67,68,69,70,71]. Similarly, angiotensin-converting enzyme inhibitors (ACEIs) and angiotensin receptor blockers (ARBs) have demonstrated positive associations with improved outcomes [67,68,69,70,71]. Beta-blockers, on the other hand, have shown inconsistent results, with most studies failing to establish a clear survival benefit, although a large study by Lindahl et al. suggested a non-significant trend toward improved outcomes [67]. Data on calcium channel blockers remain limited, with no conclusive evidence of long-term MACE reduction [68,70]. Beyond pharmacological strategies, non-drug interventions have been poorly studied. However, one investigation suggested that structured physical exercise may provide benefits for MINOCA patients [72]. Additionally, lifestyle modifications, including a healthy diet, exercise, weight control, smoking cessation, and stress management activities, and a mitigation of modifiable cardiovascular risk factors, including blood-pressure control, diabetes control, and lipid-lowering therapy, are essential to optimize long-term outcomes.

Patients diagnosed with an underlying cause for MINOCA benefit from cause-directed treatment. However, even when an etiology is suspected, the optimal management approach remains unclear.

For MINOCA cases attributed to plaque disruption, the recommended treatment includes an initial period of DAPT, followed by lifelong monotherapy alongside high-intensity statins—even in individuals with a minimal plaque burden—as well as beta-blockers and ACEIs or ARBs. Although an observational cohort study using data from the SWEDEHEART registry did not demonstrate a clear benefit of dual antiplatelet therapy, this analysis included the entire MINOCA population without distinguishing between those with confirmed plaque disruption and those with other underlying causes of MINOCA [67]. The use of a second antiplatelet agent could be justified by extrapolating data from acute myocardial infarction trials, which demonstrated an added benefit from combining a P2Y12 receptor inhibitor with aspirin [73,74]. Limited evidence exists regarding the efficacy of PCI either with or without drug-eluting stents in managing culprit lesions in MINOCA cases caused by plaque disruption. Due to this lack of robust data, expert panels and guideline committees have not endorsed PCI as a standard approach to treat MINOCA-related lesions [1,2]. Prati et al. evaluated the efficacy of dual DAPT compared with PCI with stenting in 31 patients who had plaque erosion identified via OCT. After a median follow-up period of 753 days, all participants remained symptom-free [75]. This research was the first to propose a non-invasive therapeutic alternative for individuals presenting with acute coronary syndromes despite the absence of significant arterial obstruction. The EROSION trial later reinforced these findings, demonstrating that patients conservatively managed with DAPT without undergoing PCI had a favorable clinical course [76]. At a one-year follow-up, the need for repeat revascularization was observed in only 5.7% of cases, supporting the viability of a medication-based approach in select patients with nonobstructive coronary lesions.

If vasospasm (epicardial or microvascular) is the underlying mechanism, calcium channel blockers (dihydropyridine and non-dihydropyridine) are the most effective symptomatic therapy, preventing recurrent symptoms and arrhythmias while improving mortality. High dosages of a calcium antagonist (e.g., 2 × 200 mg diltiazem daily or higher, up to 960 mg daily) or a combination of a non-dihydropyridine (such as diltiazem) with dihydropyridine calcium blockers (such as amlodipine) may be necessary. Low-dose aspirin is also effective at treating coronary vasospasm by inhibiting thromboxane-A2-mediated vasoconstriction. Nitrates may be considered as an adjunctive therapy, though the benefits of long-acting nitrates remain uncertain due to potential tolerance development [77]. Other effective therapies include nicorandil (a potassium channel activator with nitrate-like effects) and cilostazol (a phosphodiesterase type 3 inhibitor) [77]. ACEIs/ARBs are recommended, and statin therapy should be initiated if coexisting atherosclerosis is present.

In cases of microvascular dysfunction, calcium channel blockers and β-blockers have been shown to effectively reduce symptoms, whereas nitrates are less effective [78]. Several small randomized controlled trials have highlighted the effectiveness of various nontraditional antianginal treatments such as L-arginine [79], dipyridamole [80], and ranolazine [81]. Additionally, some studies suggest that combining an aldosterone antagonist with an ACEI or ARB could provide enhanced clinical outcomes in CMD [82].

For patients with SCAD, stenting carries a higher risk of complications as it may exacerbate the dissection both upstream and downstream of the affected vessel [83,84,85,86]. As a result, a conservative approach without angioplasty is typically recommended. PCI should be reserved for cases with high-risk anatomical features, such as severe lesions in the proximal left main or left anterior descending artery, a low TIMI grade, or ongoing ischemia with hemodynamic instability [87]. Coronary artery bypass grafting (CABG) is generally not an optimal treatment as grafts often fail over time due to healing processes in the grafted arteries, which may lead to competitive flow and eventual occlusion; it should be reserved for stable patients with high-risk clinical features and either an ostial LAD lesion or  ≥ 2 proximal lesions when PCI is not feasible, or left main dissection involving the LAD or left circumflex artery [88]. Long-term beta-blocker therapy is a reasonable option [87]. A large cohort study found that SCAD survivors who were treated with beta-blockers had a lower risk of recurrence [84]. As thrombi play a minimal role in SCAD pathophysiology, the use of antiplatelet therapy remains contentious. Theoretically, it increases the risk of bleeding and worsens the hematoma or dissection area; however, the intimal tear may have prothrombotic properties, making the use of a moderately potent P2Y12 inhibitor like clopidogrel a justifiable option.

The management of coronary thrombosis/embolism should focus on treating the underlying prothrombotic condition with antithrombotic and, in some cases, antiplatelet therapy. Additional treatments may be necessary for specific causes such as thrombotic thrombocytopenic purpura (TTP) or heparin-induced thrombocytopenia (HIT) [89].

For myocardial-bridge-related ischemia, beta-blockers are the first-line therapy due to their ability to prolong diastolic filling and decompress the tunneled coronary segment; among them, nebivolol appears to be particularly advantageous because of its high beta1 selectivity and potential benefits on endothelial function through beta3 receptor activation [90]. Dihydropyridine CCB and ivabradine can serve as alternatives in selected cases [91]. PCI may be an option to alleviate systolic compression in myocardial bridging, particularly for patients experiencing persistent angina despite comprehensive medical management; it is more likely to be effective in cases where the tunneled coronary segment is relatively short (<25 mm) and positioned closer to the epicardial surface (<2 mm in depth) [92]. For patients with extensive or deeply embedded myocardial bridging contributing to MINOCA, particularly when multiple stents would be required, surgical intervention, including CABG and supra-arterial myotomy (also known as “unroofing”), may be an alternative. Current evidence comparing CABG and myotomy remains limited, preventing definitive recommendations [93].

Given the gaps in evidence, further research is essential to refine treatment strategies and establish more precise management recommendations. The ongoing MINOCA-BAT (Randomized Evaluation of Beta-Blocker and ACEI/ARB Treatment in MINOCA Patients) trial aims to evaluate the effects of ACEIs/ARBs and beta-blockers versus placebos on cardiovascular outcomes and overall mortality over one year [94]. The primary outcome of MINOCA-BAT is mortality or readmission due to MI, stroke, or heart failure, and the trial is due to complete in 2025. Other studies, such as the PRIZE (Precision Medicine with Zibotentan in Microvascular Angina) trial investigating Zibotentan, may provide benefits for microvascular dysfunction management.

## 7. Sex-Related Considerations

MINOCA presents a unique challenge in cardiovascular medicine, particularly regarding sex-related differences. Women are disproportionately affected by this condition: a comprehensive meta-analysis revealed that women constitute approximately 59.5% of MINOCA cases [95], despite representing only 25% of MI-CAD cases. Women presenting with MI are more than twice as likely as men to be diagnosed with MINOCA, whereas men are more frequently diagnosed with MI-CAD.

The sex-related differences in MINOCA incidence may be attributed to distinct underlying pathophysiological mechanisms that are more prevalent in women, particularly CMD and SCAD [96,97]. CMD is significantly more prevalent women with MINOCA compared to men: in a cohort of 40 female MINOCA patients who underwent stress CMR, two-thirds displayed inducible perfusion defects, suggesting the presence of CMD [98]. Among women with stable CMD, approximately 8% exhibited myocardial scarring on CMR in the Women’s Ischemia Syndrome Evaluation (WISE) study [99]. Moreover, CMD is even more prevalent in postmenopausal women. Estrogen plays a crucial role in maintaining endothelial function by enhancing nitric oxide production and suppressing angiotensin 1 receptors and angiotensin-converting enzymes, thereby mitigating vasoconstriction induced by the renin-angiotensin system. Consequently, estrogen deficiency has been linked to increased endothelial dysfunction. Over 90% of SCAD patients are female, with a significant proportion presenting before 50 years of age [83,100]. SCAD-related MI is estimated to account for 1%–5% of all AMI cases, rising to 35% in women under 50 years of age [37,99]. Female sex hormones and pregnancy are also linked to SCAD, with most pregnancy-related MI events occurring within the first week postpartum [83]. On the other hand, men tend to present with thromboembolic or atherosclerotic causes.

Women with MINOCA frequently present with nontraditional risk factors and atypical symptoms, which can lead to misdiagnosis and suboptimal treatment. Nontraditional cardiac risk factors more prevalent or unique to women include early menopause, gestational diabetes, pregnancy-related hypertension, and systemic inflammatory disorders. These conditions contribute to endothelial dysfunction and microvascular abnormalities. Interestingly, smoking has been identified as a significant risk factor for epicardial spasm in young women, with an odds ratio of 7.7 for women smokers versus non-smokers [101]. Moreover, women are more likely to report atypical symptoms (69% compared with 58% of men), such as nausea, epigastric discomfort, dizziness, dyspnea, or profound fatigue, rather than the classic chest pain often associated with myocardial infarction. Emotional stress is a well-recognized precipitant in women [43].

An ongoing trial specifically focused on women is WARRIOR (Women’s Ischemia Trial to Reduce Events In Non-Obstructive CAD). This multicenter prospective study is designed to assess the effectiveness of intensive medical therapy, consisting of high-intensity statins and ACEIs or ARBs, against usual care in 4422 symptomatic women with a diagnosis of INOCA (anginal symptoms and/or signs of ischemia and no obstructive coronary artery disease) in the United States in terms of MACE and mortality over 3 years [102]. This research is particularly important as increasing the inclusion of women in clinical trials related to INOCA/MINOCA and cardiovascular disease in general is a critical step toward achieving equitable care.

## 8. Conclusions

MINOCA is a complex and heterogeneous clinical entity requiring a systematic diagnostic approach to identify its underlying mechanisms. Advances in imaging techniques, particularly cardiac magnetic resonance and intracoronary assessments, have improved diagnostic accuracy and risk stratification. Despite growing awareness, standardized treatment protocols remain limited and are often extrapolated from management strategies of obstructive coronary artery disease. The significant sex-related differences in MINOCA underscore the need for personalized therapeutic approaches. Future research, including ongoing clinical trials, is essential to refine management strategies and improve outcomes for patients with MINOCA.

## Data Availability

Not applicable.
